# The potential of mechanistic information organised within the AOP framework to increase regulatory uptake of the developmental neurotoxicity (DNT) *in vitro* battery of assays

**DOI:** 10.1016/j.reprotox.2021.06.006

**Published:** 2021-08

**Authors:** Magdalini Sachana, Catherine Willett, Francesca Pistollato, Anna Bal-Price

**Affiliations:** a Environment Health and Safety Division, Environment Directorate, Organisation for Economic Co-Operation and Development (OECD), 75775, Paris Cedex 16, France; b Humane Society International, 1255 23rd Street NW, Washington, DC, 20037, USA; c European Commission Joint Research Centre (JRC), Ispra, Italy

**Keywords:** *In vitro* developmental neurotoxicity testing, Regulatory purposes, Adverse outcome pathways, Integrated approaches to testing and assessment, Neurodevelopmental disorders

## Abstract

A major challenge in regulatory developmental neurotoxicity (DNT) assessment is lack of toxicological information for many compounds. Therefore, the Test Guidelines programme of the Organisation for Economic Cooperation and Development (OECD) took the initiative to coordinate an international collaboration between diverse stakeholders to consider integration of alternative approaches towards improving the current chemical DNT testing. During the past few years, a series of workshops was organized during which a consensus was reached that incorporation of a DNT testing battery that relies on *in vitro* assays anchored to key neurodevelopmental processes should be developed. These key developmental processes include neural progenitor cell proliferation, neuronal and oligodendrocyte differentiation, neural cell migration, neurite outgrowth, synaptogenesis and neuronal network formation, as well key events identified in the existing Adverse Outcome Pathways (AOPs). AOPs deliver mechanistic information on the causal links between molecular initiating event, intermediate key events and an adverse outcome of regulatory concern, providing the biological context to facilitate development of Integrated Approaches to Testing and Assessment (IATA) for various regulatory purposes. Developing IATA case studies, using mechanistic information derived from AOPs, is expected to increase scientific confidence for the use of *in vitro* methods within an IATA, thereby facilitating regulatory uptake. This manuscript summarizes the current state of international efforts to enhance DNT testing by using an *in vitro* battery of assays focusing on the role of AOPs in informing the development of IATA for different regulatory purposes, aiming to deliver an OECD guidance document on use of *in vitro* DNT battery of assays that include *in vitro* data interpretation.

## Highlights

• Current *in vivo* DNT testing for regulatory purposes is not effective.
• *In vitro* assays anchored to key neurodevelopmental processes are available.
• Development of Adverse Outcome Pathways is required to increase mechanistic understanding of DNT effects.
• DNT Integrated Approaches to Testing and Assessment for various regulatory purposes should be developed.
• The OECD Guidance Document on use of *in vitro* DNT battery of assays is currently under development.

## 1 Introduction

Developmental neurotoxicity (DNT) is defined as the adverse functional and morphological effects induced by chemical exposure on the developing nervous system of offspring that may arise from exposure in utero and during early life [1]. These effects can include motor, sensory and cognitive deficits [1,2]. DNT testing for assessing chemical safety is currently performed by using the available EPA and the Organisation for Economic Cooperation and Development (OECD) Test Guidelines [2,3] that rely on rodent models and measure alterations at histological, physiological and neurobehavioral levels in pups following perinatal exposure [[4], [5], [6]]. The DNT testing requirements in the different chemical regulatory sectors across the various OECD member countries are mostly linked to triggers derived from other systemic testing related to neurotoxicity and/or developmental toxicity or endocrine disruption. This has historically limited the generation of detailed DNT animal data to those chemicals where testing was triggered [7].

Other factors that impeded the broader use of DNT Test Guidelines are the complexity of the *in vivo* tests, the difficulty in interpreting results and the need of significant resources, contributing to the environmental release of chemicals that haven’t been ever tested for their DNT potential [8]. In a number of workshops and scientific papers, regulators, academic and industrial partners agreed on the need for developing more efficient and potentially more effective testing strategies to assess the DNT potential of chemicals, one that relies on more recent biological understanding and technological methods relevant to DNT [[9], [10], [11], [12], [13], [14], [15], [16], [17], [18], [19], [20]]. Notably, a workshop that brought together experts and regulators in the field, jointly organised by the Organisation for Economic Cooperation and Development (OECD) and the European Food Safety Authority (EFSA) in 2016, led to a consensus that some *in vitro* DNT assays [17] could be used immediately for screening of chemicals and prioritization, and, following further harmonization through OECD, eventual use for hazard identification and characterization [17,21]. In response to the outcome and recommendations of this workshop [17,21], in 2017, the OECD convened an international expert group to develop a guidance document on the application of *in vitro* assays within integrated approaches for testing and assessment (IATA) to address DNT and to assist with data interpretation of these assays and IATA.

Two critical aspects are important when developing guidance for internationally recognised non-animal-based approaches to address challenging endpoints such as DNT: a) the anchoring of *in vitro* assays to mechanistic knowledge, and b) the integration of multiple information sources not restricted to data only derived from the DNT *in vitro* battery (DNT-IVB). For this reason, the OECD chemical safety programme has been working over the last ten years towards the development of IATA that rely on combination of multiple layers of information (*e.g.*, epidemiological information, existing *in vivo*, *in vitro*, *in silico* and non-mammalian *in vivo* data) and that can be supported by mechanistic knowledge organised according the adverse outcome pathway (AOP) framework.

The AOP framework was introduced and adopted by the OECD to allow structured collection, organisation and evaluation of existing scientific knowledge and that could ultimately facilitate regulatory decision-making [[22], [23], [24]]. This knowledge is collected and assembled taking into account all levels of biological organisation and covering a wide range of data that derive from molecular, cellular, clinical and epidemiological studies. AOPs explicitly capture the linkage of measurable and essential biological effects (key events) to an adverse outcome (AO). The molecular initiating event (MIE) can be triggered by chemical exposure, and the priority adverse outcomes are of regulatory importance, such as DNT. One of the main contributions of the OECD AOP programme and its supporting community is to build a knowledgebase (https://aopkb.oecd.org/ and https://aopwiki.org/) that stores mechanistic information relevant to adverse health effects that would support development of mechanistically-informed and human-relevant alternative assays to be used for toxicity testing within the OECD Test Guidelines programme [22]. Another important contribution is the mechanistic-based guidance for mapping data from various sources, in particular for the integration of information from a battery of *in vitro* assays and even beyond using the IATA framework [[24], [25], [26]]. In this context, AOP-informed IATA will play a pivotal role in shifting emphasis from traditional, current regulatory DNT toxicity testing that is entirely based on animals, to more tailored, hypothesis-driven predictive approaches, taking into account mechanistic information at various levels of biological organization built in AOPs relevant to DNT.

This paper briefly summarises the current status of the OECD regulatory and scientific effort to develop guidance on the development and implementation of a DNT-IVB, leading to improved and faster chemical testing for this specific endpoint. The paper also discusses the considerations for regulatory uptake of the DNT-IVB and the overall role of the mechanistic information captured in AOPs, which should be incorporated into the IATA framework.

## 2 Current status of the international effort to develop an OECD guidance document on *in vitro* DNT battery

DNT is one of several toxicological (as well as medical) areas of high concern whose biological underpinnings are complex due to multiple neurodevelopmental processes which take place during brain development [27]. Key neurodevelopmental processes include commitment and differentiation to neural progenitor cells followed by their proliferation, migration, differentiation into various neuronal and glial subtypes, synaptogenesis, synaptic pruning, myelination, and neuronal network formation with neuronal and glial maturation [15,28]. These processes are tightly regulated across different brain structures at specific stages of brain development. These spatio-temporal specificities open up very distinct developmental windows of susceptibility towards the same chemical exposure. It is well documented that disruption of these key neurodevelopmental processes by developmental neurotoxicants may modify neuronal/glial cell birth, structure and function leading to alterations in neuroanatomy, neurophysiology, neurochemistry, and neurobehavior, resulting in a variety of adverse outcomes [17,29].

Therefore, an *in vitro* DNT testing battery that permits quantitative evaluation of these key neurodevelopmental processes following exposure to chemical(s) was proposed in the OECD/EFSA workshop [17,30] and considered by the OECD project as a basis for formulating a guidance document. A fundamental assumption of this approach is that any disturbance of these key neurodevelopmental processes can be potentially reflected through DNT-related phenotypes *in vivo*. Disturbances of key neurodevelopmental processes are considered as important biomarkers of potential DNT-induced chemical effects at the cellular level. The readiness of the existing *in vitro* DNT assays for various regulatory application has been recently evaluated and, depending on the problem formulation, these assays will be incorporated into an IATA accordingly [28].

Currently, rodent and human neuronal and glial cell models are available which can deliver a range of reliable *in vitro* assays and data that permit quantitative evaluation (*via* concentration-response relationships) of the impact of a compound on various stages of brain development. Recently there has been an emphasis on using human *in vitro* neuronal cultures derived from neural progenitor cells (NPCs) as they are self-renewable and can be differentiated into several neuronal and glial cell types [28,31]. Human primary NPCs derived from brain foetal tissues or from induced pluripotent stem cells (hiPSCs) differentiated into mixed populations of neuronal/glial cells are mainly used, as they mimic *in vitro* critical brain developmental processes, including proliferation, migration and neuronal, astrocyte and oligodendrocyte differentiation as well as neuronal network formation and maturation measured by evaluation of synaptogenesis, myelination and neuronal network activity [15,28,32]. A range of *in vitro* test methods exists for studying these critical neurodevelopmental processes, whereas *in vitro* assays permitting evaluation of glia-specific processes, such as oligodendrocytes differentiation, myelin formation, radial and astroglia development, differentiation, microglia interaction/activation, and functional responses to chemical exposure are still not well developed or missing. Taking into consideration these gaps in the current DNT-IVB, CEFIC has recently published a call titled “*Expansion of a regulatory accepted in vitro testing battery for developmental neurotoxicity (DNT) evaluation*” (http://cefic-lri.org/request-for-proposals/lri-aimt11-expansion-of-a-regulatory-accepted-in-vitro-testing-battery-for-developmental-neurotoxicity-dnt-evaluation/), aiming to deliver the missing *in vitro* assays that could be incorporated into the existing DNT-IVB.

Another step forward towards the use of alternative approaches for DNT for regulatory purposes is the recently published EFSA report on the implementation and interpretation of an *in vitro* testing battery for the assessment of DNT [33]. In this project, human cell-based DNT test methods with high readiness underwent a fit-for-purpose evaluation taking into consideration the exposure scheme, the assay and analytical endpoint(s) and the classification model. This *in vitro* testing battery, consisting of 5 test methods measuring 10 DNT-specific endpoints and 9 viability/cytotoxicity-related parameters, was challenged with 119 chemicals for which rich toxicological information was available. The applied DNT testing strategy is promising since its performance demonstrated a sensitivity of 82.7 % and a specificity of 88.2 %, even with the known battery gaps.

All these research efforts aim to contribute towards the development of an OECD guidance on the interpretation and use of data derived from the DNT-IVB that can be applied to address relevant regulatory questions. Additional experimental work that will feed in this international effort is expected from the US Environmental Protection Agency (US EPA) that will be made available through the US EPA’s CompTox Chemicals Dashboard (https://comptox.epa.gov/dashboard/chemical_lists/TOXCAST) and is expected to be finalised in 2021.

**Fig. 1:**
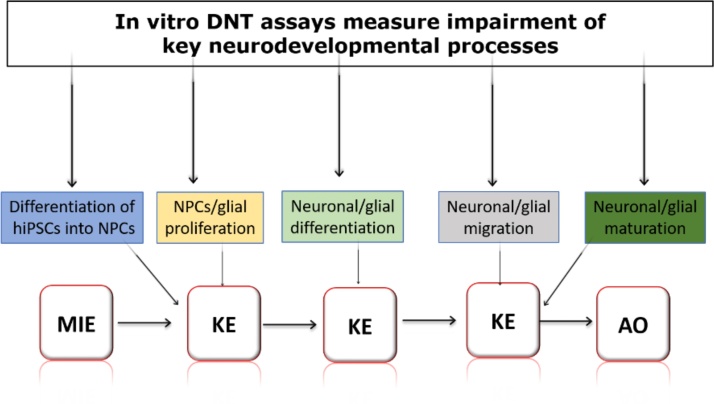
Simplified mechanistic knowledge captured in AOPs speeds up development of relevant *in vitro* DNT assays to be further incorporated in the existing DNT-IVB and insures coverage of key DNT mechanisms. Schematic representation of impaired key neurodevelopmental processes defined as key events (KEs) in DNT AOPs (hiPSCs: human induced pluripotent stem cells; NPCs: Neural Precursor cells; MIE: Molecular Initiating Event; AO: Adverse Outcome).

*In vitro* assays that are not yet part of the DNT-IVB to be included in the OECD guidance can also be valuable and complementary to the *in vitro* battery and the regulatory decisions related to DNT. However, these additional *in vitro* assays that, for example, might come out from CEFIC initiatives and other similar funding opportunities would need to demonstrate validity by providing the scientific basis, reproducibility and predictive capacity of the new test methods including testing of accepted positive and negative DNT chemicals. Such a list of reference compounds will be part of the DNT-IVB guidance document and include chemicals tested in the current battery [33]. New *in vitro* assays that are based on the understanding of molecular and cellular mechanisms captured in AOPs can facilitate the evolution and increase regulatory acceptance of the DNT-IVB (Fig. 1).

The objective of the OECD project, overseen by an OECD Expert Group on DNT, is to deliver a guidance document that will describe a framework to facilitate the regulatory application of DNT *in vitro* data from the battery through the linkage to AOPs and by applying the IATA framework. The intention is not to replace the *in vivo* Test Guidelines, but to illustrate through the guidance that there are a number of regulatory problem formulations, for which data from the DNT-IVB can be used for decision-making. A certain number of these problem formulations will be captured in case studies and published together with the OECD guidance to demonstrate the practical use of the DNT-IVB.

## 3 Rational for developing an AOP-informed IATA for *in vitro* DNT testing

Design of testing strategies that would sufficiently cover the breadth of biological processes involved in neurodevelopmental disorders requires inclusion of a battery of *in vitro* assays, using a range of different types of test methods, that capture alterations of the key neurodevelopmental processes (ideally in a quantitative manner). To achieve this goal, it is useful to capture neurodevelopmental processes as AOPs, since the AOP framework allows compilation, structured organisation and evaluation of biological information demonstrating the causal links between steps in each process or pathway. In the AOP framework, these pathways are structured as beginning with the molecular initiating event (MIE), the initial perturbation triggered by a stressor (*e.g.*, chemical exposure), followed by subsequent perturbations of downstream key events (KEs) at cellular, tissue, organ and organism levels, representing a cascade of toxicity pathways potentially resulting in adverse outcomes (AO) of regulatory relevance [23,34]. Individual pathways converge or diverge to form biological networks through shared key events: common key events (CKEs) shared among several key pathways would be priority candidates for the development of assays to query that shared network of AOPs. Therefore, application of the AOP framework offers the biological context for IATA development, increasing scientific confidence in use of mechanistic knowledge for regulatory decision-making.

Development of AOP-informed IATA starts with problem formulation (including a description of the chemical, regulatory framework, type of decision), followed by gathering all available data relevant to the specific question, including available *in vivo*, epidemiological information and any data coming from *in silico*, *in vitro* and other non-animal tests [24,28,35]. The existing information is evaluated through a weight of evidence (WoE) approach to decide whether it is adequate for a specific regulatory conclusion. Already at this stage, the AOP framework can be applied to organise and review the existing information in a structured and tailored manner guided by the KE and key event relationship (KER) descriptions and can support the WoE evaluation based on expert judgment.

**Fig. 2:**
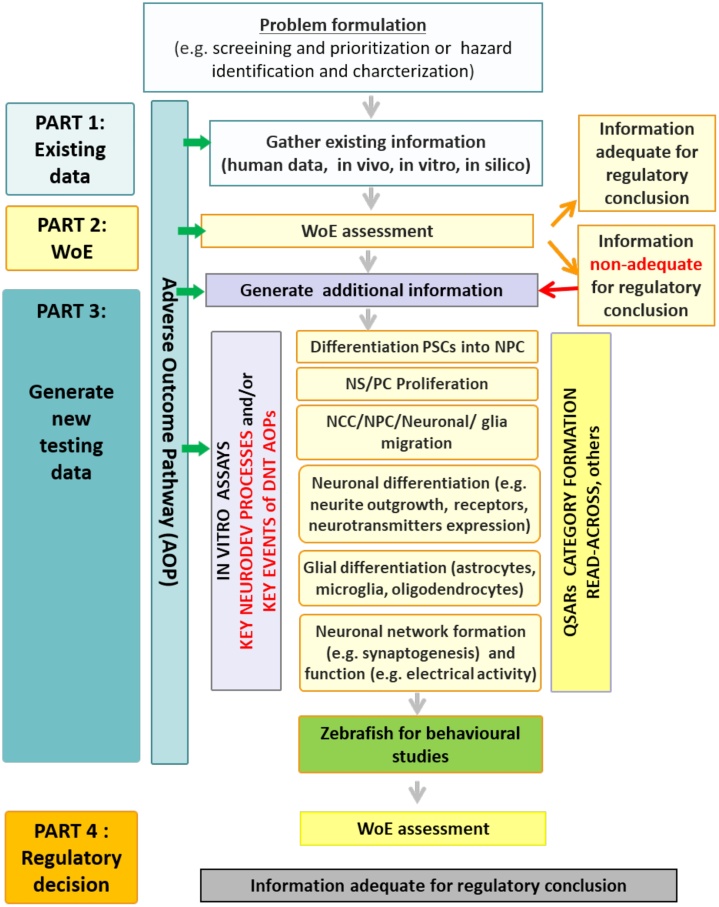
**Building blocks of an AOP-informed IATA specific for DNT which integrates multiple sources of information.** KEs and KERs defined in AOP guide reviewing of existing information and if necessary, the targeted generation of new data using a battery of *in vitro* assays anchored to key neurodevelopmental processes and key events identified in AOP(s). If required, some behavioural tests can be performed using zebrafish and targeted animal *in vivo* studies. Combination of these approaches (including QSARs, read across *etc.*) will depend on problem formulation (screening and prioritization or hazard identification/characterization). (WoE: Weight of Evidence; PSCs: Pluripotent Stem Cells; NPCs: Neural Precursor Cells; QSARs: Quantitative Structure Activity Relationship). (Modified Fig. 2 from Bal-Price et al., 2018 [28]).

If new data are needed to inform the decision with the necessary certainty, the AOP network can guide experimental work to deliver required information. As a first tier, it is recommended that *in vitro* assays should be anchored to selected set of CKEs [15,19] identified in AOPs relevant to DNT [[36], [37], [38]], delivering missing data (Fig. 2). Moreover, data from *in vitro* assays that allow an evaluation of the key neurodevelopmental processes specific for brain development but not yet defined as KEs in AOPs (*e.g.*, cell proliferation, migration, differentiation, *etc.*) if needed, should also be incorporated into such IATA.

Based on the fit-for-purpose principle, the combination of different *in vitro* DNT assays and other non-testing methods will vary. For instance, our current level of understanding supports the application of an IATA based entirely on a DNT-IVB of assays for chemical screening and prioritization, since these decisions tolerate a higher level of uncertainty. IATA for hazard identification and characterization should potentially include highly standardized *in vitro* assays and potentially be combined with read across and QSARs, driven always by the problem formulation. In the case of a decision bearing on risk assessment, information on exposure and ADME will need to be incorporated into the IATA. Zebrafish is the only relatively high-throughput non-mammalian animal model to study DNT that could potentially complement the DNT-IVB and provide additional data to be considered under IATA. A subgroup of the OECD DNT expert group is currently working towards standardization of zebrafish protocols and testing of significant number of compounds in 4–6 different laboratories.

Taking into consideration these various regulatory applications (prioritization/screening, hazard identification/characterization and risk assessment), 17 *in vitro* DNT assays were evaluated based on the (semi)-quantitative analysis of their readiness with respect to various use since none of these assays had previously been validated following classical EURL-ECVAM validation process. The scoring results suggest that several assays are currently at high readiness levels. Therefore, suggestions are made on how DNT *in vitro* assays may be assembled into an IATA, depending on the problem formulation [28].

The DNT-IVB (Fig. 2) proposed in the OECD guidance document is supported by currently available DNT AOPs [19,36,37]. For example, “*learning and memory impairment in children/decrease of cognitive function*” is identified as the most common AO among the currently available DNT AOPs (AOP Wiki; AOP 13: https://aopwiki.org/aops/13; AOP 54: https://aopwiki.org/aops/54; AOP 42: https://aopwiki.org/aops/42). These AOPs are triggered by various MIEs that lead to different early KEs, but converge on CKEs close to AOs including neuronal differentiation, synaptogenesis or neuronal network formation and function [32]. Interestingly, these CKEs overlap with key neurodevelopmental processes which can be quantitatively measured using *in vitro* test methods based on human mixed neuronal glial cultures [17,33]. Therefore, the assays that permit quantitative *in vitro* evaluation of these CKEs are the first choice candidates for inclusion in IATA DNT-IVB. The current low number of DNT-AOPs concerning neurodevelopmental KEs should not prohibit the use of the battery, but might foster further development of DNT AOPs.

## 4 Development of case studies to support the OECD DNT guidance document

Case studies are in development to illustrate the integration of *in vitro* data derived from the testing battery together with other information sources by applying the IATA framework. These case studies will support the OECD DNT guidance by providing valuable context-specific examples. All case studies begin with problem formulations designed to address a number of regulatory needs by applying targeted DNT *in vitro* testing. In addition, the IATA case studies aim to elucidate how mechanistic understanding of chemical-induced DNT that is guided in most cases by AOPs can support the required decision-making. The case studies will cover the following scenarios: a) evaluation of DNT alerts that derive from computational models, b) screening and prioritization of chemical libraries with no DNT data, c) design of screening strategies to test small chemical classes, some of which have *in vivo* DNT data and others not, and d) individual chemical hazard assessment (*e.g.*, in case where no DNT information exist, when the DNT data are contradictory or when a chemical is re-evaluated in light of new evidence).

It is expected that with the release of the guidance and the systematic use of the battery, additional experience will be gained and knowledge will be gathered to effectively address not only the above-mentioned but also additional regulatory scenarios. New IATA case studies that might be developed after completion of the OECD guidance document can further enrich the regulatory application of the DNT-IVB by shedding more light on DNT mechanistic understanding and by incorporating additional *in vitro* assays. Finally, the broader use of the DNT-IVB will generate data that would allow the building of new AOPs and the expansion of the existing AOP network.

## 5 Mechanistic information provided by an AOP-driven IATA will improve chemical assessment

AOP networks relevant to neurotoxicity have recently been published [[37], [38], [39]], and several of the identified KEs correspond with key neurodevelopmental processes. One of the first AOP networks described perturbation of thyroid hormone signalling which can lead to abnormal brain development, cognitive impairments, and other adverse outcome in humans and wildlife [39].

The AOP network published by Spinu et al. [38] covers AOPs submitted to the AOP Wiki and includes both DNT and adult neurotoxicity, since the MIE and/or some KEs could occur during the neurodevelopment stage and hence could also be potentially relevant to DNT. This AOP network shows that currently the most common AO is defined as “*impairment of learning and memory in children/decrease of cognitive function*” and can be triggered by diverse MIEs, including binding of antagonists to NMDA receptor (AOP ID 12 and 13), binding of agonists to NMDA receptor (AOP ID 48), inhibition of the thyroid peroxidase (TPO) (AOP ID 42) or inhibition of the sodium/iodide symporter (NIS) (AOP ID 54). These MIEs trigger different early KEs but, closer to AO, they converge on common KEs (CKEs), such as alteration of synaptogenesis and neuronal network function. The AOP network described by Li et al. identified additional CKEs including mitochondrial dysfunction, cell injury/neurodegeneration, altered differentiation of neural stem/precursor cells into neurons and glia cells, and decreased T4 levels in neuronal tissue [37].

*In vitro* batteries of assays anchored to CKEs which are triggered by multiple MIEs may serve as simplification for the activation of multiple AOPs. By choosing those CKEs as testing priorities, the number of assays can be reduced while still covering multiple AOPs covering a variety of MIEs triggered by different classes of chemicals. This rational was applied for evaluation of DNT effects induced by mixtures of chemicals using a set of *in vitro* assays, anchored to CKEs of several AOPs with the common AO of *“learning and memory impairment in children”* [32,40].

Although endocrine disruption-based DNT AOPs are outside of the scope of the OECD guidance document under development, the thyroid disruption-triggered AOPs (*e.g.*, AOP 54 in the AOP Wiki) contain KEs that are relevant to DNT and should be included for consideration when designing a battery of *in vitro* assays.

The available AOP network covers only a very limited range of potential neurodevelopmental disorders. Therefore, further efforts are needed to develop AOPs, especially for AOs with increasing prevalence, including some of those mentioned above: autism spectrum disorder (ASD); attention-deficit/hyperactivity disorder (ADHD); intellectual disability (also known as mental retardation); conduct disorders; cerebral palsy; impairments in vision and hearing; motor disorders including developmental coordination disorder and stereotypic movement disorder; communication, speech and language disorders; foetal alcohol spectrum disorder, open neural tube defects, *etc.* These are complex diseases, where complex mechanisms and pathways are likely to be involved.

Having a comprehensive network of DNT AOPs that covers most major neurodevelopmental diseases will allow the identification of a more comprehensive set of CKEs. It would also clarify to what extent these diseases are covered by the existing AOPs, CKEs and associated *in vitro* test batteries, which processes are still not being covered and for which processes assays should be developed.

The current *in vivo* test guidelines do not predict neurodevelopmental disorders [41], nor do they address or provide information on mechanisms, but rather measure the chemicals’ potential to induce DNT (in rodents). It follows that the advancements in mechanistic understanding of key pathways involved in developmental neurobiology and neuropathology, combined with knowledge coming from studies on chemical exposure such as *Neurosome* (https://www.neurosome.eu/) and the Japan Environment and Children’s Study (https://www.env.go.jp/chemi/ceh/en/index.html), when organised using the AOP framework, will further improve our ability to both understand the effects of and to better regulate chemical exposure through the use of alternative approaches for DNT testing.

## 6 Overview of what is covered and what is missing in current AOPs relevant to DNT

Neurodevelopmental disorders are multifaceted conditions characterized by impairments in cognition, learning disabilities, speech and language disorders, poor social development, behaviour and/or motor skills resulting from abnormal brain development. Intellectual disability, communication disorders, ASD, ADHD, epilepsy and schizophrenia fall also under the umbrella of neurodegenerative diseases (NDDs) where a gene-environmental interplay is involved.

Moreover, many symptoms are not unique to a single NDD, and several NDDs have clusters of symptoms in common. For example, impaired social cognition is common to ASD and schizophrenia. Furthermore, NDDs may have diverse pathophysiology that underlies similar clinical phenotypes, or conversely, diverse clinical outcomes may result from similar pathophysiology. For example, ASD is a neurodevelopmental disease with an increasing incidence and is more prevalent in males [[42], [43], [44]]. However, it is now clear that ASD is an umbrella term for multiple disorders with overlapping clinical symptoms, suggesting that there are shared and unique pathophysiological mechanisms which have yet to be identified. Therefore, developing an AOP for autism (and for many other neurodevelopmental disorders) is challenging as there is a general lack of understanding of the potential multifactorial MIE(s) that are causally responsible for triggering KEs resulting in complex ASD symptoms. Nevertheless, difficulties in identifying MIEs for many DNT AOPs may not hinder the application of these AOPs to design relevant testing strategies.

Currently, there are only a few AOPs relevant to DNT or adult neurotoxicity available that are already endorsed by Working Party of National Co-ordinators of the Test Guidelines Programme and the Working Party on Hazard Assessment (https://aopkb.oecd.org/ and https://aopwiki.org) (AOP ID 12, 13, 54, 42, 48, 3, 10). Others have been submitted to the AOP Wiki (*e.g.*, AOPs 17, 134, 8, 152, 281,260, 161, 160, 279, 230, 231) or published in peer review journals [37,45] but they are still at early stages of development. There are also a fair number of AOP proposals that are relevant to DNT and have been included in the OECD AOP workplan (https://www.oecd.org/chemicalsafety/testing/projects-adverse-outcome-pathways.htm) and are expected to be submitted to the AOP-Wiki and reviewed in the near future [[46], [47], [48], [49]].

Although there are only 7 fully OECD endorsed AOPs, the existing network of AOPs covers already biological space critical to several adverse outcomes and identifies CKEs that can contribute to both hypothesis generation and the design of IATA for DNT assessment. A few DNT AOPs have been developed referring to impairment of learning and memory/cognitive damage in children ([19,39], AOP 13, AOP 48, AOP17, AOP 54, AOP 42, AOP 152; all in the AOP Wiki: https://aopwiki.org) triggered by diverse MIEs including the interaction of chemicals with specific receptors and enzymes. There is also an AOP resulting in neurological dysfunction and hearing loss (AOP 8) triggered by upregulation of thyroid hormone catabolism (https://aopwiki.org/wiki/index.php/Aop:8), and a few AOPs published describing neurological and cognitive impairment triggered by interferences with thyroid hormone synthesis, metabolism and transport [46].

The analyses of modes-of-action of DNT compounds as well as of the pathways contributing to impairment of neurodevelopmental processes showed that multiple pathways involved in neurodevelopmental disorders merge into most of these processes. These are summarized in Table 1 and support the concept of using neurodevelopmental processes and major signalling pathways as anchors for building *in vitro* testing battery because these pathways were identified based on triggers for human DNT (*e.g.*, by human DNT compounds or mutations causing neurodevelopmental syndromes) [17,21].

**Table 1:** Overview of *in vitro* assays covering key neurodevelopmental processes and major signalling pathways involved in neurodevelopmental disorders (it includes some pathway published by E. Fritsche [17]).

Key neuro- developmental processes	*In vitro* assay available Yes/No	Signaling pathways involved in impairment of neurodevelopmental processes	References
Differentiation of Human Pluripotent Stem Cells towards Neural Stem Cells	Yes	Wnt/ β-catenin signaling,; dorsal forebrain neural progenitor cells (PAX6 + OTX1/2+); telencephalon (FOXG1), the ventral telencephalon (LHX8, LHX6, NKX2-1, DLX1, and DLX5), the hindbrain (HOXA2 and HOXB2), and the dorsal telencephalon (cortex) (EMX1, EMX2, and EOMES)	[61,62]
		Extracellular matrix (ECM), CREB activity/phosphorylation	[63]
		Notch sygnalling (Mash1, Ngn2); NSCs maintenance and differentiation	[64]
Neural precursor Cells and Neural Crest Cells: Neurogenesis and Proliferation	Yes	SH-group maintenance; Redox balance; Histone acetylation/deacetylation; Prostaglandin signalling; mTORC1-STAT3, mTOR-GSK3ß	[65]
		activation RTKPI3K-AKT signalling; PGE2 – wnt signalling; BDNF-ERK-CREB, retinoic acid signaling	[66]
		mTORC1 and mTORC2	[67]
		TH signalling	[68,69]
		GSK3B	[70]
		Forskolin, Indomethacin induced increase in cAMP and activity of Protein Kinases A/B	[66,71,72,73]
		Neurotrophins, RA induced signalling through MAPK/ERK and PI3K/Akt activity	[74,75,76]
Neural Progenitor Cell Neuronal and Radial glia migration	Yes	PLCgamma1, GDNF-RET, BDNF/TrkB, PDGFR, FGFR, mTORC1 signalling; MAP kinase and Reelin-Dab pathways, PGE2 – wnt signalling; PLCgamma1-dependent calcium release with activation of PKC	[77,78]
		N-cadherin, RhoA activation.	[79]
		Integrin alpha3beta1, reelin	[80]
Neural Migration	Yes	PDGFR-PLCγ1; BDNF/TrkB activates MAPK and PI3K pathways and PLCγ1-dependent IP3-mediated calcium release; GDNF-RET-mediated activation CaMKII; PDGFR-mediated activation of PLCγ1 with production of intracellular DAG gradient, ERC activation, retinoic acid signaling, RET and JNK dependent migration.	[78]
		Reelin, ApoER2 and VLDLR	[81]
		BDNF⁄TrkB interaction, PI3-K, MAP-K	[82]
		Cdk5, Dab1, Rac1, FAK, Ras, Src, and PI3K	[83]
		Calm1, Gria1 (GluA1) and Camk4 (calmodulin-signaling network), Hdac2 and Hsbp1 (Akt1-DNA transcription network), Vav3 and Ppm1a	[84]
Differentiation and maturation of Neural Stem Cells into distinct neuronal cell types	Yes	mTORC1/C2, prostaglandin, Histone acetylation/deacetylation, miRNA-9, miRNA-17-92 cluster, miRNA-124, Notch signalling, Wnt, BDNF, retinoic acid signaling.	[64,67]
		SHH and Notch pathway activation (for the maintenance of neural rosette cells)	[85]
		Wnt/β-catenin pathway, β -catenin/TCF, neurogenin 1	[86]
		aPKC, PRKCI and PRKCZ	[87]
		Wnt factors signal through canonical, β-catenin pathway, planar cell polarity pathway and calcium pathway	[88]
		Neuron maturation relevant functional modules in protein-protein interaction (PPI) network	[89]
Synaptogenesis	Yes	NMDA-receptor activation, BDNF-Trk signalling, calcium signalling, inositol metabolism, Phospholipase D activity with generation of phosphatidic acid; BDNF-ERK-CREB/decreased activity/phosphorylation	[65]
		ProSAP1/Shank2, MaGuK family, including SAP90/PSD-95, Munc13, RIMs, ERC/CAST, Piccolo/Aczonin, and Bassoon, Shank1, Neuroligin, and GKAP	[90]
		Wnt signalling through β-catenin-dependent pathways	[91]
Apoptosis	Yes	*N*-methyl-d-aspartate (NMDA) receptors, the retinoic acid receptors, brain-derived neurotrophic factor (BDNF), insulin-like growth factor 1 (IGF-I), and basic fibroblast growth factor (bFGF)	[92]
		miR-132	[93]
		Caspase-dependent cell death, AIF, Bcl-2 and its related family member Bcl-xL	[94,95]
Radial glia proliferation and migration	Yes	miRNA-9	[96]
		FGF-MAPK cascade, SHH, PTEN/AKT, PDGF pathways, and proteins such as INSM, GPSM2, ASPM, TRNP1, ARHGAP11B, PAX6, and HIF1α	[97]
		FGF-2	[98]
		Notch, ErbB, and fibroblast growth factor	[99]
Differentiation into Astrocytes	Yes	mTORC1-STAT3 signalling, Notch signalling; miRNA-124; mTORC2 signalling	[67]
		Activation of MAPK/ERK and increase in JAK/STAT	[100,101,102]
		Induction of gp130 receptors for JAK/STAT activity	[103,104,105]
		Fibronectin, BMP signalling	[106]
		Astrocytic expression of NFIAA/B and GLAST-1, S100β is an astrocyte progenitor marker, GFAP, GS, EAAT1 and EAAT2, AQP-4, GS, GLT-1, astrocytic early stage marker: ALDH1	[107]
		LIF, STAT3, RA	[108]
		CSL-dependent Notch signalling pathway	[109]
Differentiation into Oligodendrocytes	Yes	Extracellular matrix (ECM), fibroblast growth factor (FGF)-2, retinoic acid (RA), EGF morphogenetic protein antagonists such as noggin, neurotrophic factors such as neurotrophin (NT)-3 and ciliary neurotrophic factor, with or without EGF	[110,111,112,113,114,115]
		TH signalling	[68,69]
		Signalling through RA and p38 MAPK pathways	[116,117,118]
		Induction of transcription factors by Notch and Shh	[119,120,121]
Differentiation into Microglia	No	CD45^−^/c-KIT^−^/CX3CR1^+^ cells in a PU-1, IRF-8, and colony stimulating factor 1 (CSF-1R), RFD7^+^) and monocyte-associated markers (UCHM1^+^)	[122]
		Signaling through fractalkine receptor (e.g., CX_3_CR1), cell survival factor (CSF) 1-receptor (CSF1-R), the transcription factors, PU.1 (SPl1) and interferon regulatory factor 8 (IRF8)	[123,124]
Differentiation into Schwann cells	Yes	Neregulin-1, LPA-induced increase in cAMP by activation of GPR44 and GPR126	[125,126]
		Neurotrophins induction of specific transcription factors by PI3K/Akt	[127,128]
Outgrowth of dendrites & axons	Yes	FGFR-mediated PLCγ1- PKC activation with subsequent IP3 and AA formation; NCAM/FGFR- PLCγ1 with action on small GTPases, such as Rho A, Rac1, or Cdc42; cytoskeleton maintenance; CREB and BDNF signling, MAPK activation	[129]
		RYR sensitization, PIP metabolism, BDNF-ERK-CREB: Prostaglandins, cyclooxygenases, EP receptors; BDNF, ERK-CREB	[78]
		TH signaling,	[68,69]
		BDNF-ERK-CREB	[65]
Axon guidance	Yes	PLCγ1-dependent calcium release with activation of PKC, BDNF, TRPC3/6 channels, IP3	[78]
		Plexin B, EphA, ephrin B and Robo, regulate the Rho family of GTPases	[130]
		Rho family small guanosine triphosphatases (GTPases)	[131]
		TH signaling	[68,69]
Neuronal subtypes	Yes	Interference with calcium signalling; miRNA-124; AKT signaling and miR-9/9*	[132]
		ASE chemosensory neurons lys-6 COG-1, miR-273, DIE-1 induction of ASEL identity	[133,134]
		Anterior–posterior axis: miR-9 Hes1 (homologs)	[135,136]
		Cortex: miR-9, FoxG1, several other targets	[137,138]
		Olfactory bulb: Pax 6, miR-7°, *etc*.	[139]
		Midbrain: miR-135a Lmx1b. Delimiting the DV extent of the dopaminergic progenitor pool	[140]
		Midbrain–hindbrain boundary: miR-9, Fgfr1, Canopy, Fgf8, Her5, Her9	[141]
		Retina: miR-129, miR-155, miR-214, miR-222, Xotx2, Xvsx1, let-7, miR-125, miR-9 Ptrg, Lin28b	[142,143]
		Spinal cord miR-17-3p Olig2, miR-196, Hoxb8, miR-9 FoxP1, OC1	[144,145,146,147]
Neuronal network formation (synaptogenesis) and synaptic plasticity	Yes	Inositol metabolism; PIP(2&3); PI3K, Prostaglandins (cyclooxygenases, EP receptors); TH signalling; EphrinA1/EphA4 - PLCγ1 (structural synaptic plasticity); BDNF/TrkB - PLCγ1, BDNF, ERK-CREB; BDNF-ERK-CREB (GABA maturation)	[78,148,149]
		TH signaling	[68,69]
		BDNF-ERK-CREB	[65]

Further insight into chemical modes of action could be facilitated by tools being developed by the US National Toxicology Program Interagency Center for the Evaluation of Alternative Toxicological Methods (NICEATM), including the Integrated Chemical Environment (ICE) and the Developmental NeuroToxicity Data Integration and Visualization Enabling Resource (DNT-DIVER). ICE provides an interactive dashboard for accessing curated in silico, *in vitro* and *in vivo* data from multiple sources as well as a set of computational workflows to assist with chemical activity characterization [50]. DNT-DIVER compares results from a limited number of DNT-related assays for a limited number of chemicals; the potential utility of this tool will increase as assays and curated data are added [51].

The most studied DNT compound is lead and indeed, there is already an AOP endorsed by OECD working parties of the chemical safety programme where lead is described as a trigger of the MIE defined as “binding of antagonists to NMDA receptor [52]. One of the mechanisms triggered by lead is inhibition of BDNF-Trk signalling, causing aberrant dendritic morphology, decreased synaptogenesis and decreased neuronal network formation and function. Impairment of these key neurodevelopmental processes may lead to decreased learning and memory processes in children [52,53]. During different stages of brain development, BDNF-associated signalling pathways are also affected by exposure to ethanol, another well-established DNT compound [[54], [55], [56]].

Synaptogenesis is also affected by exposure to chlorpyrifos [57] and organophosphates [58,59], inducing disturbance of cAMP-CREB signalling pathway, resulting in impaired axonal and dendritic differentiation.

Separate studies are required to link potential DNT compounds with perturbed signalling pathways described in Table 1. Such an exercise would be beneficial to establish mechanistic understanding of DNT effects induced by environmental chemicals or drugs as potential DNT compounds.

Ideally, all biological pathways leading to neurological and developmental neurological pathologies would be known and described in the form of AOPs. Without knowing the full extent of coverage, it is possible that the existing AOPs cover only limited DNT-relevant biological space and mechanisms of concern. Therefore, it is desirable to gather further mechanistic understanding of neurological diseases captured as AOPs, to explicitly describe and link to major neurodevelopmental adverse outcomes not yet covered. Further development of AOPs will inform about the mechanistic space that is potentially not assessed by the current proposed DNT-IVB and identify KEs as anchors for development of additional *in vitro* assays.

As mentioned earlier, development of AOPs for complex neurodevelopmental disorders such as ASD or ADHD is challenging because of (1) a general lack of understanding of the contribution of biology, including genetic and environmental factors to these disorders, (2) a lack of identification of specific pathways that might be triggered, including potential MIEs and causally linked KEs that lead to these outcomes. The molecular and cellular mechanisms of many neurodevelopmental disorders are largely unknown and in some cases, features/symptoms of these diseases are overlapping since they are not specific for one particular disease. Therefore, the complex aetiology and limited mechanistic understanding of neurodevelopmental disorders make identification of KEs (which would be linked linearly and supported by empirical data described in KERs) extremely difficult. Additional difficulty arises from the dynamic processes of brain development, which can be affected differently by the same chemical, depending on the window of exposure and the susceptibility of specific brain regions and various neuronal or glial subtypes at different times during development.

**Fig. 3:**
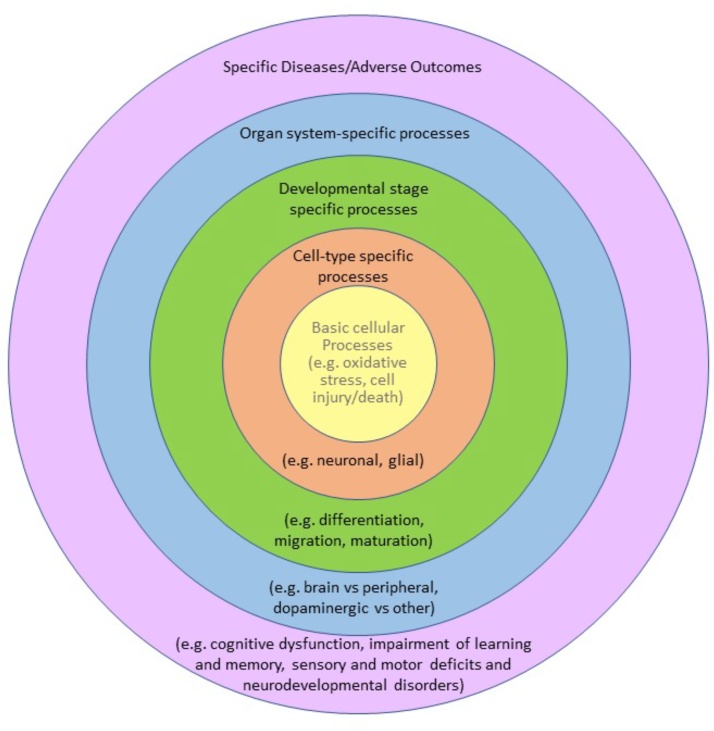
Biological space represented as concentric spheres of increasing specificity, containing examples specific to the developing nervous system.

One advantage of focussing on AOPs describing basic neurodevelopmental processes is to make sure that the testing battery covers these fundamental processes, increasing the likelihood that any neurodevelopmental effect will be captured, even if it cannot yet be explicitly described in an AOP and linked to critical KEs or AOs. Another advantage of the AOP framework is that, as biological information is added and AOP networks evolve, testing strategies could be designed for different purposes or levels of specification (*e.g.*, Fig. 3). A testing battery that covers CKEs based on several cellular processes (*e.g.*, mitochondria impairment, oxidative stress, dysfunction of ion channels, receptors, changes in neurotransmitter synthesis/release, increased proinflammatory cytokines production, *etc.*) could be used as a precautionary screen, irrespective of identification of a particular pathway or AO. A battery of more specific CKEs could be used to identify more specific pathways or developmental stages (*e.g.*, synaptogenesis, neuronal network formation and function or cell-type-specific differentiation or migration), or network or pathway specific KEs could then be used to identify affected pathways with increasing specificity (*e.g.*, specific to impairment of learning or memory, or neurodevelopmental disorder-specific). The current DNT-IVB included in the OECD guidance document is based on assays that mostly cover this biological space but is not considered exhaustive.

For diseases not covered by current AOPs, such as ASD and ADHD *etc.*, it is important to increase the mechanistic understanding to make sure the underlying mechanisms can be covered by available testing approaches. Therefore, when developing future DNT-related AOPs, the systematic organization of available information and the identification of critical knowledge gaps are equally important. *In vitro* assays anchored to KEs can deliver relevant molecular and cellular information which would contribute to better mechanistic understanding of pathways involved, especially when using human-cell based test methods, contributing to further AOP development relevant to neurodevelopmental disorders.

## 7 Conclusions

The AOP framework provides the opportunity for explicitly describing both the common and unique molecular features of the various developmental stages of the nervous system. Developing AOP networks reflecting the complexity of the key neurodevelopmental processes that are anchored to phenotypic adverse outcomes seems a challenging task. However, emerging experimental data can provide significant empirical evidence and support to AOP building and reveal better understanding of chemicals’ involvement in manifestation of DNT relevant adverse outcomes. In parallel, epidemiological studies complemented by ‘omics’ generated data from patient samples (*e.g.*, cerebrospinal fluid) or information gathered from functional MRI could potentially, in the near future, reveal reliable biomarkers of effects that could further confirm and provide stronger evidence supporting the DNT-relevant AOPs. Most importantly, studies that provide quantitative human data would identify factors that modify potential outcomes; these factors could then be described quantitatively in KER for eventual modelling, for example to predict susceptible individuals.

Activities for the development of DNT relevant IATAs and the use of DNT-IVB can draw from the mechanistic information that is structurally stored in individual AOPs or AOP networks. Developed IATA case studies can also lead to the generation of new mechanistic data and hence development of novel AOPs, as it has happened recently in an EFSA DNT project where the trigger-specific (deltamethrin) AOP network has been created as part of the IATA case study [33,60], and will soon be published.

AOP-supported IATA can address various regulatory and non-regulatory problem formulations that range from chemical prioritisation to risk assessment. Developing IATA case studies using focused problem formulations supported by mechanistic information from AOPs, with clear recognition of uncertainties involved, can ultimately increase the uptake and gain support from the regulatory community.

## Disclaimers

The opinions expressed and arguments employed herein are those of the authors and do not necessarily reflect the official views of the OECD or of the governments of its member countries.

## Declaration of Competing Interest

The authors declare that they have no known competing financial interests or personal relationships that could have appeared to influence the work reported in this paper.

## References

[1] OECD (2018). OECD Guidelines for the Testing of Chemicals. Section 4: Health Effects. Test No. 443: Extended One Generation Reproductive Toxicity Study. https://www.oecd-ilibrary.org/environment/test-no-443-extended-one-generation-reproductive-toxicity-study_9789264185371-en.

[2] US. EPA (1998). Health Effects Guidelines OPPTS 870.6300 Developmental Neurotoxicity Study. https://nepis.epa.gov/Exe/ZyNET.exe/P100IRWO.txt?ZyActionD=ZyDocument%26Client=EPA%26Index=1995%20Thru%201999%26Docs=%26Query=%26Time=%26EndTime=%26SearchMethod=1%26TocRestrict=n%26Toc=%26TocEntry=%26QField=%26QFieldYear=%26QFieldMonth=%26QFieldDay=%26UseQField=%26IntQFieldOp=0%26ExtQFieldOp=0%26XmlQuery=%26File=D%3A%5CZYFILES%5CINDEX%20DATA%5C95THRU99%5CTXT%5C00000034%5CP100IRWO.txt%26User=ANONYMOUS%26Password=anonymous%26SortMethod=h%7C-%26MaximumDocuments=1%26FuzzyDegree=0%26ImageQuality=r75g8/r75g8/x150y150g16/i425%26Display=hpfr%26DefSeekPage=x%26SearchBack=ZyActionL%26Back=ZyActionS%26BackDesc=Results%20page%26MaximumPages=1%26ZyEntry=1.

[3] OECD (2007). OECD Guidelines for the Testing of Chemicals. Section 4: Health Effects. Test No. 426: Developmental Neurotoxicity Study. https://www.oecd-ilibrary.org/environment/test-no-426-developmental-neurotoxicity-study_9789264067394-en.

[4] Makris S.L., Raffaele K., Allen S., Bowers W.J., Hass U., Alleva E., Calamandrei G., Sheets L., Amcoff P., Delrue N., Crofton K.M. (2009). A retrospective performance assessment of the developmental neurotoxicity study in support of OECD test guideline 426. Environ. Health Perspect..

[5] Tsuji R., Crofton K.M. (2012). Developmental neurotoxicity guideline study: issues with methodology, evaluation and regulation. Congenit. Anom. (Kyoto).

[6] Tohyama C. (2016). Developmental neurotoxicity test guidelines: problems and perspectives. J. Toxicol. Sci..

[7] Smirnova L., Hogberg H.T., Leist M., Hartung T. (2014). Developmental neurotoxicity - challenges in the 21st century and in vitro opportunities. ALTEX.

[8] Crofton K.M., Mundy W.R., Shafer T.J. (2012). Developmental neurotoxicity testing: a path forward. Congenit. Anom. (Kyoto).

[9] Lein P., Locke P., Goldberg A. (2007). Meeting report: alternatives for developmental neurotoxicity testing. Environ. Health Perspect..

[10] Coecke S., Goldberg A.M., Allen S., Buzanska L., Calamandrei G., Crofton K., Hareng L., Hartung T., Knaut H., Honegger P., Jacobs M., Lein P., Li A., Mundy W., Owen D., Schneider S., Silbergeld E., Reum T., Trnovec T., Monnet-Tschudi F., Bal-Price A. (2007). Workgroup report: incorporating in vitro alternative methods for developmental neurotoxicity into international hazard and risk assessment strategies. Environ. Health Perspect..

[11] Kuegler P.B., Zimmer B., Waldmann T., Baudis B., Ilmjarv S., Hescheler J., Gaughwin P., Brundin P., Mundy W., Bal-Price A.K., Schrattenholz A., Krause K.H., van Thriel C., Rao M.S., Kadereit S., Leist M. (2010). Markers of murine embryonic and neural stem cells, neurons and astrocytes: reference points for developmental neurotoxicity testing. ALTEX.

[12] Crofton K.M., Mundy W.R., Lein P.J., Bal-Price A., Coecke S., Seiler A.E., Knaut H., Buzanska L., Goldberg A. (2011). Developmental neurotoxicity testing: recommendations for developing alternative methods for the screening and prioritization of chemicals. ALTEX.

[13] Kadereit S., Zimmer B., van Thriel C., Hengstler J.G., Leist M. (2012). Compound selection for in vitro modeling of developmental neurotoxicity. Front. Biosci. Landmark Ed..

[14] Bal-Price A.K., Coecke S., Costa L., Crofton K.M., Fritsche E., Goldberg A., Grandjean P., Lein P.J., Li A., Lucchini R., Mundy W.R., Padilla S., Persico A.M., Seiler A.E., Kreysa J. (2012). Advancing the science of developmental neurotoxicity (DNT): testing for better safety evaluation. ALTEX.

[15] Bal-Price A., Pistollato F., Sachana M., Bopp S.K., Munn S., Worth A. (2018). Strategies to improve the regulatory assessment of developmental neurotoxicity (DNT) using in vitro methods. Toxicol. Appl. Pharmacol..

[16] Mundy W.R., Padilla S., Breier J.M., Crofton K.M., Gilbert M.E., Herr D.W., Jensen K.F., Radio N.M., Raffaele K.C., Schumacher K., Shafer T.J., Cowden J. (2015). Expanding the test set: Chemicals with potential to disrupt mammalian brain development. Neurotoxicol. Teratol..

[17] Fritsche E. (2017). Report on Integrated Testing Strategies for the Identification and Evaluation of Chemical Hazards Associated With the Developmental Neurotoxicity (DNT), to Facilitate Discussions at the Joint EFSA/OECD Workshop on DNT Series on Testing and Assessment No. 261.

[18] Schmidt B.Z., Lehmann M., Gutbier S., Nembo E., Noel S., Smirnova L., Forsby A., Hescheler J., Avci H.X., Hartung T., Leist M., Kobolak J., Dinnyes A. (2017). In vitro acute and developmental neurotoxicity screening: an overview of cellular platforms and high-throughput technical possibilities. Arch. Toxicol..

[19] Bal-Price A., Crofton K.M., Leist M., Allen S., Arand M., Buetler T., Delrue N., FitzGerald R.E., Hartung T., Heinonen T., Hogberg H., Bennekou S.H., Lichtensteiger W., Oggier D., Paparella M., Axelstad M., Piersma A., Rached E., Schilter B., Schmuck G., Stoppini L., Tongiorgi E., Tiramani M., Monnet-Tschudi F., Wilks M.F., Ylikomi T., Fritsche E. (2015). International STakeholder NETwork (ISTNET): creating a developmental neurotoxicity (DNT) testing road map for regulatory purposes. Arch. Toxicol..

[20] Fritsche E., Grandjean P., Crofton K.M., Aschner M., Goldberg A., Heinonen T., Hessel E.V.S., Hogberg H.T., Bennekou S.H., Lein P.J., Leist M., Mundy W.R., Paparella M., Piersma A.H., Sachana M., Schmuck G., Solecki R., Terron A., Monnet-Tschudi F., Wilks M.F., Witters H., Zurich M.G., Bal-Price A. (2018). Consensus statement on the need for innovation, transition and implementation of developmental neurotoxicity (DNT) testing for regulatory purposes. Toxicol. Appl. Pharmacol..

[21] OECD (2017). Report of the OECD/EFSA Workshop on Developmental Neurotoxicity (DNT): the Use of Non-animal Test Methods for Regulatory Purposes, Series on Testing and Assessment No. 261.

[22] Delrue N., Sachana M., Sakuratani Y., Gourmelon A., Leinala E., Diderich R. (2016). The adverse outcome pathway concept: a basis for developing regulatory decision-making tools. Altern. Lab. Anim..

[23] OECD (2018). Users’ Handbook Supplement to the Guidance Document for Developing and Assessing Adverse Outcome Pathways. https://www.oecd-ilibrary.org/environment/users-handbook-supplement-to-the-guidance-document-for-developing-and-assessing-adverse-outcome-pathways_5jlv1m9d1g32-en.

[24] OECD (2017). Guidance Document for the Use of Adverse Outcome Pathways in Developing Integrated Approaches to Testing and Assessment (IATA). http://www.oecd.org/chemicalsafety/guidance-document-for-the-use-of-adverse-outcome-pathways-in-developing-integrated-approaches-to-testing-and-assessment-iata-44bb06c1-en.htm.

[25] Sachana M., Leinala E. (2017). Approaching chemical safety assessment through application of Integrated approaches to testing and assessment: combining mechanistic information derived from adverse outcome pathways and alternative methods. Appl. In Vitro Toxicol..

[26] Sakuratani Y., Horie M., Leinala E. (2018). Integrated approaches to testing and assessment: OECD activities on the development and use of adverse outcome pathways and case studies. Basic Clin. Pharmacol. Toxicol..

[27] Stiles J., Jernigan T.L. (2010). The basics of brain development. Neuropsychol. Rev..

[28] Bal-Price A., Hogberg H.T., Crofton K.M., Daneshian M., FitzGerald R.E., Fritsche E., Heinonen T., Hougaard Bennekou S., Klima S., Piersma A.H., Sachana M., Shafer T.J., Terron A., Monnet-Tschudi F., Viviani B., Waldmann T., Westerink R.H.S., Wilks M.F., Witters H., Zurich M.G., Leist M. (2018). Recommendation on test readiness criteria for new approach methods in toxicology: exemplified for developmental neurotoxicity. ALTEX.

[29] Fritsche E., Barenys M., Klose J., Masjosthusmann S., Nimtz L., Schmuck M., Wuttke S., Tigges J. (2018). Development of the concept for stem cell-based developmental neurotoxicity evaluation. Toxicol. Sci..

[30] Fritsche E., Crofton K.M., Hernandez A.F., Hougaard Bennekou S., Leist M., Bal-Price A., Reaves E., Wilks M.F., Terron A., Solecki R., Sachana M., Gourmelon A. (2017). OECD/EFSA workshop on developmental neurotoxicity (DNT): the use of non-animal test methods for regulatory purposes. ALTEX.

[31] Fritsche E., Barenys M., Klose J., Masjosthusmann S., Nimtz L., Schmuck M., Wuttke S., Tigges J. (2018). Current availability of stem cell-based in vitro methods for developmental neurotoxicity (DNT) testing. Toxicol. Sci..

[32] Pistollato F., de Gyves E.M., Carpi D., Bopp S.K., Nunes C., Worth A., Bal-Price A. (2020). Assessment of developmental neurotoxicity induced by chemical mixtures using an adverse outcome pathway concept. Environ. Health.

[33] Masjosthusmann S., Blum J., Bartmann K., Dolde X., Holzer A.K., Lynn E.H.K., Stürzl C., Förster N., Dönmez A., Klose J., Pahl M., Waldmann T., Bendt J.K.F., Suciu I., Hübenthal U., Mosig A., Marcel L., Fritsche E. (2020). Establishment of an a Priori Protocol for the Implementation and Interpretation of an In‐vitro Testing Battery for the Assessment of Developmental Neurotoxicity.

[34] Edwards S.W., Tan Y.M., Villeneuve D.L., Meek M.E., McQueen C.A. (2016). Adverse outcome pathways-organizing toxicological information to improve decision making. J. Pharmacol. Exp. Ther..

[35] Patlewicz G., Kuseva C., Kesova A., Popova I., Zhechev T., Pavlov T., Roberts D.W., Mekenyan O. (2014). Towards AOP application--implementation of an integrated approach to testing and assessment (IATA) into a pipeline tool for skin sensitization. Regul. Toxicol. Pharmacol..

[36] Bal-Price A., Meek M.E.B. (2017). Adverse outcome pathways: application to enhance mechanistic understanding of neurotoxicity. Pharmacol. Ther..

[37] Li J., Settivari R., LeBaron M.J., Marty M.S. (2019). An industry perspective: a streamlined screening strategy using alternative models for chemical assessment of developmental neurotoxicity. Neurotoxicology.

[38] Spinu N., Bal-Price A., Cronin M.T.D., Enoch S.J., Madden J.C., Worth A.P. (2019). Development and analysis of an adverse outcome pathway network for human neurotoxicity. Arch. Toxicol..

[39] Noyes P.D., Friedman K.P., Browne P., Haselman J.T., Gilbert M.E., Hornung M.W., Barone S., Crofton K.M., Laws S.C., Stoker T.E., Simmons S.O., Tietge J.E., Degitz S.J. (2019). Evaluating chemicals for thyroid disruption: opportunities and challenges with in vitro testing and adverse outcome pathway approaches. Environ. Health Perspect..

[40] Davidsen N., Lauvås A.J., Myhre O., Ropstad E., Carpi D., Gyves E.M., Berntsen H.F., Dirven H., Paulsen R.E., Bal-Price A., Pistollato F. (2021). Exposure to human relevant mixtures of halogenated persistent organic pollutants (POPs) alters neurodevelopmental processes in human neural stem cells undergoing differentiation. Reprod. Toxicol. (Elmsford, N.Y.).

[41] Paparella M., Bennekou S.H., Bal-Price A. (2020). An analysis of the limitations and uncertainties of in vivo developmental neurotoxicity testing and assessment to identify the potential for alternative approaches. Reprod. Toxicol. (Elmsford, N.Y.).

[42] Lyall K., Croen L., Daniels J., Fallin M.D., Ladd-Acosta C., Lee B.K., Park B.Y., Snyder N.W., Schendel D., Volk H., Windham G.C., Newschaffer C. (2017). The changing epidemiology of autism Spectrum disorders. Annu. Rev. Public Health.

[43] McDonald M.E., Paul J.F. (2010). Timing of increased autistic disorder cumulative incidence. Environ. Sci. Technol..

[44] Cheroni C., Caporale N., Testa G. (2020). Autism spectrum disorder at the crossroad between genes and environment: contributions, convergences, and interactions in ASD developmental pathophysiology. Mol. Autism.

[45] Costa A.P., Steffgen G., Samson A.C. (2017). Expressive incoherence and Alexithymia in autism Spectrum disorder. J. Autism Dev. Disord..

[46] Bal-Price A., Lein P.J., Keil K.P., Sethi S., Shafer T., Barenys M., Fritsche E., Sachana M., Meek M.E.B. (2017). Developing and applying the adverse outcome pathway concept for understanding and predicting neurotoxicity. Neurotoxicology.

[47] Barenys M., Reverte I., Masjosthusmann S., Gómez-Catalán J., Fritsche E. (2020). Developmental neurotoxicity of MDMA. A systematic literature review summarized in a putative adverse outcome pathway. Neurotoxicology.

[48] Chen H., Chidboy M.A., Robinson J.F. (2020). Retinoids and developmental neurotoxicity: utilizing toxicogenomics to enhance adverse outcome pathways and testing strategies. Reprod. Toxicol. (Elmsford, N.Y.).

[49] Klose J., Tigges J., Masjosthusmann S., Schmuck K., Bendt F., Hübenthal U., Petzsch P., Köhrer K., Koch K., Fritsche E. (2021). TBBPA targets converging key events of human oligodendrocyte development resulting in two novel AOPs. ALTEX.

[50] Bell S., Abedini J., Ceger P., Chang X., Cook B., Karmaus A.L., Lea I., Mansouri K., Phillips J., McAfee E., Rai R., Rooney J., Sprankle C., Tandon A., Allen D., Casey W., Kleinstreuer N. (2020). An integrated chemical environment with tools for chemical safety testing. Toxicol. In Vitro.

[51] NTP (2018). Data Release: Developmental NeuroToxicity Data Integration and Visualization Enabling Resource (DNT-DIVER).

[52] Sachana M., Rolaki A., Bal-Price A. (2018). Development of the Adverse Outcome Pathway (AOP): chronic binding of antagonist to N-methyl-d-aspartate receptors (NMDARs) during brain development induces impairment of learning and memory abilities of children. Toxicol. Appl. Pharmacol..

[53] Stansfield K.H., Pilsner J.R., Lu Q., Wright R.O., Guilarte T.R. (2012). Dysregulation of BDNF-TrkB signaling in developing hippocampal neurons by Pb(2+): implications for an environmental basis of neurodevelopmental disorders. Toxicol. Sci..

[54] Light K.E., Ge Y., Belcher S.M. (2001). Early postnatal ethanol exposure selectively decreases BDNF and truncated TrkB-T2 receptor mRNA expression in the rat cerebellum. Brain Res. Mol. Brain Res..

[55] Shojaei S., Ghavami S., Panjehshahin M.R., Owji A.A. (2015). Effects of ethanol on the expression level of various BDNF mRNA isoforms and their encoded protein in the Hippocampus of adult and embryonic rats. Int. J. Mol. Sci..

[56] Yu Y., Xu D., Cheng S., Zhang L., Shi Z., Qin J., Zhang Z., Wang H. (2020). Prenatal ethanol exposure enhances the susceptibility to depressive behavior of adult offspring rats fed a high‑fat diet by affecting BDNF‑associated pathway. Int. J. Mol. Med..

[57] Howard A.S., Bucelli R., Jett D.A., Bruun D., Yang D., Lein P.J. (2005). Chlorpyrifos exerts opposing effects on axonal and dendritic growth in primary neuronal cultures. Toxicol. Appl. Pharmacol..

[58] Adigun A.A., Seidler F.J., Slotkin T.A. (2010). Disparate developmental neurotoxicants converge on the cyclic AMP signaling cascade, revealed by transcriptional profiles in vitro and in vivo. Brain Res..

[59] Verma S.K., Raheja G., Gill K.D. (2009). Role of muscarinic signal transduction and CREB phosphorylation in dichlorvos-induced memory deficits in rats: an acetylcholine independent mechanism. Toxicology.

[60] EFSA (2021). Public Consultation on a Draft Scientific Opinion on Development of Integrated Approaches to Testing and Assessment (IATA) on Developmental Neurotoxicity (DNT) Risk Assessment. https://www.efsa.europa.eu/en/consultations/call/public-consultation-draft-scientific-opinion-development.

[61] Moya N., Cutts J., Gaasterland T., Willert K., Brafman D.A. (2014). Endogenous WNT signaling regulates hPSC-derived neural progenitor cell heterogeneity and specifies their regional identity. Stem Cell Rep..

[62] Strano A., Tuck E., Stubbs V.E., Livesey F.J. (2020). Variable outcomes in neural differentiation of human PSCs arise from intrinsic differences in developmental signaling pathways. Cell Rep..

[63] Brafman D.A. (2015). Generation, expansion, and differentiation of human pluripotent stem cell (hPSC) derived neural progenitor cells (NPCs). Methods Mol. Biol..

[64] Imayoshi I., Shimojo H., Sakamoto M., Ohtsuka T., Kageyama R. (2013). Genetic visualization of notch signaling in mammalian neurogenesis. Cell. Mol. Life Sci.: CMLS.

[65] Ehrlich D.E., Josselyn S.A. (2016). Plasticity-related genes in brain development and amygdala-dependent learning. Genes Brain Behav..

[66] Hevner R.F. (2015). Brain overgrowth in disorders of RTK-PI3K-AKT signaling: a mosaic of malformations. Semin. Perinatol..

[67] Lee D.Y. (2015). Roles of mTOR signaling in brain development. Exp. Neurobiol..

[68] Patel J., Landers K., Li H., Mortimer R.H., Richard K. (2011). Thyroid hormones and fetal neurological development. J. Endocrinol..

[69] de Escobar G.M., Obregón M.J., del Rey F.E. (2004). Maternal thyroid hormones early in pregnancy and fetal brain development, best practice & research. Clin. Endocrinol. Metabol..

[70] Gonzalez Malagon S.G., Lopez Muñoz A.M., Doro D., Bolger T.G., Poon E., Tucker E.R., Adel Al-Lami H., Krause M., Phiel C.J., Chesler L., Liu K.J. (2018). Glycogen synthase kinase 3 controls migration of the neural crest lineage in mouse and Xenopus. Nat. Commun..

[71] Jang S., Cho H.H., Cho Y.B., Park J.S., Jeong H.S. (2010). Functional neural differentiation of human adipose tissue-derived stem cells using bFGF and forskolin. BMC Cell Biol..

[72] Kim S.S., Choi J.M., Kim J.W., Ham D.S., Ghil S.H., Kim M.K., Kim-Kwon Y., Hong S.Y., Ahn S.C., Kim S.U., Lee Y.D., Suh-Kim H. (2005). cAMP induces neuronal differentiation of mesenchymal stem cells via activation of extracellular signal-regulated kinase/MAPK. Neuroreport.

[73] Kompisch K.M., Lange C., Steinemann D., Skawran B., Schlegelberger B., Müller R., Schumacher U. (2010). Neurogenic transdifferentiation of human adipose-derived stem cells? A critical protocol reevaluation with special emphasis on cell proliferation and cell cycle alterations. Histochem. Cell Biol..

[74] Cargnello M., Roux P.P. (2011). Activation and function of the MAPKs and their substrates, the MAPK-activated protein kinases. Microbiol. Mol. Biol. Rev..

[75] Janesick A., Wu S.C., Blumberg B. (2015). Retinoic acid signaling and neuronal differentiation. Cell. Mol. Life Sci..

[76] Kaplan D.R., Miller F.D. (2000). Neurotrophin signal transduction in the nervous system. Curr. Opin. Neurobiol..

[77] Lafourcade C.A., Lin T.V., Feliciano D.M., Zhang L., Hsieh L.S., Bordey A. (2013). Rheb activation in subventricular zone progenitors leads to heterotopia, ectopic neuronal differentiation, and rapamycin-sensitive olfactory micronodules and dendrite hypertrophy of newborn neurons. J. Neurosci..

[78] Kang D.S., Yang Y.R., Lee C., Kim S., Ryu S.H., Suh P.G. (2016). Roles of phosphoinositide-specific phospholipase Cγ1 in brain development. Adv. Biol. Regul..

[79] Jinnou H., Sawada M., Kawase K., Kaneko N., Herranz-Pérez V., Miyamoto T., Kawaue T., Miyata T., Tabata Y., Akaike T., García-Verdugo J.M., Ajioka I., Saitoh S., Sawamoto K. (2018). Radial glial fibers promote neuronal migration and functional recovery after neonatal brain injury. Cell Stem Cell.

[80] Belvindrah R., Graus-Porta D., Goebbels S., Nave K.A., Müller U. (2007). Beta1 integrins in radial glia but not in migrating neurons are essential for the formation of cell layers in the cerebral cortex. J. Neurosci..

[81] Hirota Y., Nakajima K. (2017). Control of neuronal migration and aggregation by reelin signaling in the developing cerebral cortex. Front. Cell Dev. Biol..

[82] Chiaramello S., Dalmasso G., Bezin L., Marcel D., Jourdan F., Peretto P., Fasolo A., De Marchis S. (2007). BDNF/ TrkB interaction regulates migration of SVZ precursor cells via PI3-K and MAP-K signalling pathways. Eur. J. Neurosci..

[83] Khodosevich K., Monyer H. (2011). Signaling in migrating neurons: from molecules to networks. Front. Neurosci..

[84] Khodosevich K., Seeburg P.H., Monyer H. (2009). Major signaling pathways in migrating neuroblasts. Front. Mol. Neurosci..

[85] Elkabetz Y., Panagiotakos G., Al Shamy G., Socci N.D., Tabar V., Studer L. (2008). Human ES cell-derived neural rosettes reveal a functionally distinct early neural stem cell stage. Genes Dev..

[86] Hirabayashi Y., Itoh Y., Tabata H., Nakajima K., Akiyama T., Masuyama N., Gotoh Y. (2004). The Wnt/beta-catenin pathway directs neuronal differentiation of cortical neural precursor cells. Development (Cambridge, England).

[87] Hapak S.M., Rothlin C.V., Ghosh S. (2019). aPKC in neuronal differentiation, maturation and function. Neuronal Signal..

[88] Rosso S.B., Inestrosa N.C. (2013). WNT signaling in neuronal maturation and synaptogenesis. Front. Cell. Neurosci..

[89] He Z., Yu Q. (2018). Identification and characterization of functional modules reflecting transcriptome transition during human neuron maturation. BMC Genomics.

[90] Liebau S., Vaida B., Storch A., Boeckers T.M. (2007). Maturation of synaptic contacts in differentiating neural stem cells. Stem Cells.

[91] He C.W., Liao C.P., Pan C.L. (2018). Wnt signalling in the development of axon, dendrites and synapses. Open Biol..

[92] Kumar A., LaVoie H.A., DiPette D.J., Singh U.S. (2013). Ethanol neurotoxicity in the developing cerebellum: underlying mechanisms and implications. Brain Sci..

[93] Chen D., Hu S., Wu Z., Liu J., Li S. (2018). The role of MiR-132 in regulating neural stem cell proliferation, differentiation and neuronal maturation. Cell. Physiol. Biochem..

[94] Moskowitz M.A., Lo E.H. (2003). Neurogenesis and apoptotic cell death. Stroke.

[95] van Leyen K., Lee S.R., Moskowitz M.A., Lo E.H., Janigro D. (2006). Neurogenesis and apoptotic cell death. The Cell Cycle in the Central Nervous System.

[96] Petri R., Malmevik J., Fasching L., Åkerblom M., Jakobsson J. (2014). miRNAs in brain development. Exp. Cell Res..

[97] Penisson M., Ladewig J., Belvindrah R., Francis F. (2019). Corrigendum: Genes and Mechanisms Involved in the Generation and Amplification of Basal Radial Glial Cells. Front. Cell. Neurosci..

[98] Gregg C., Weiss S. (2003). Generation of functional radial glial cells by embryonic and adult forebrain neural stem cells. J. Neurosci..

[99] Ever L., Gaiano N. (2005). Radial’ glial’ progenitors: neurogenesis and signaling. Curr. Opin. Neurobiol..

[100] Callihan P., Ali M.W., Salazar H., Quach N., Wu X., Stice S.L., Hooks S.B. (2014). Convergent regulation of neuronal differentiation and Erk and Akt kinases in human neural progenitor cells by lysophosphatidic acid, sphingosine 1-phosphate, and LIF: specific roles for the LPA1 receptor. ASN Neuro.

[101] Michelucci A., Bithell A., Burney M.J., Johnston C.E., Wong K.Y., Teng S.W., Desai J., Gumbleton N., Anderson G., Stanton L.W., Williams B.P., Buckley N.J. (2016). The neurogenic potential of astrocytes is regulated by inflammatory signals. Mol. Neurobiol..

[102] Wang T., Yuan W., Liu Y., Zhang Y., Wang Z., Zhou X., Ning G., Zhang L., Yao L., Feng S., Kong X. (2015). The role of the JAK-STAT pathway in neural stem cells, neural progenitor cells and reactive astrocytes after spinal cord injury. Biomed. Rep..

[103] Ernst M., Jenkins B.J. (2004). Acquiring signalling specificity from the cytokine receptor gp130. Trends in genetics: TIG.

[104] McManus M.F., Chen L.C., Vallejo I., Vallejo M. (1999). Astroglial differentiation of cortical precursor cells triggered by activation of the cAMP-dependent signaling pathway. J. Neurosci..

[105] Paco S., Hummel M., Plá V., Sumoy L., Aguado F. (2016). Cyclic AMP signaling restricts activation and promotes maturation and antioxidant defenses in astrocytes. BMC Genomics.

[106] Pous L., Deshpande S.S., Nath S., Mezey S., Malik S.C., Schildge S., Bohrer C., Topp K., Pfeifer D., Fernández-Klett F., Doostkam S., Galanakis D.K., Taylor V., Akassoglou K., Schachtrup C. (2020). Fibrinogen induces neural stem cell differentiation into astrocytes in the subventricular zone via BMP signaling. Nat. Commun..

[107] Chandrasekaran A., Avci H.X., Leist M., Kobolák J., Dinnyés A. (2016). Astrocyte differentiation of human pluripotent stem cells: new tools for neurological disorder research. Front. Cell. Neurosci..

[108] Asano H., Aonuma M., Sanosaka T., Kohyama J., Namihira M., Nakashima K. (2009). Astrocyte differentiation of neural precursor cells is enhanced by retinoic acid through a change in epigenetic modification. Stem Cells.

[109] Ge W., Martinowich K., Wu X., He F., Miyamoto A., Fan G., Weinmaster G., Sun Y.E. (2002). Notch signaling promotes astrogliogenesis via direct CSL-mediated glial gene activation. J. Neurosci. Res..

[110] Hu B.Y., Du Z.W., Zhang S.C. (2009). Differentiation of human oligodendrocytes from pluripotent stem cells. Nat. Protoc..

[111] Kang S.M., Cho M.S., Seo H., Yoon C.J., Oh S.K., Choi Y.M., Kim D.W. (2007). Efficient induction of oligodendrocytes from human embryonic stem cells. Stem cells (Dayton, Ohio).

[112] Li Y., Liu M., Yan Y., Yang S.T. (2014). Neural differentiation from pluripotent stem cells: the role of natural and synthetic extracellular matrix. World J. Stem Cells.

[113] Sher F., Balasubramaniyan V., Boddeke E., Copray S. (2008). Oligodendrocyte differentiation and implantation: new insights for remyelinating cell therapy. Curr. Opin. Neurol..

[114] Sundberg M., Hyysalo A., Skottman H., Shin S., Vemuri M., Suuronen R., Narkilahti S. (2011). A xeno-free culturing protocol for pluripotent stem cell-derived oligodendrocyte precursor cell production. Regen. Med..

[115] Sundberg M., Skottman H., Suuronen R., Narkilahti S. (2010). Production and isolation of NG2+ oligodendrocyte precursors from human embryonic stem cells in defined serum-free medium. Stem Cell Res..

[116] Chew L.J., Coley W., Cheng Y., Gallo V. (2010). Mechanisms of regulation of oligodendrocyte development by p38 mitogen-activated protein kinase. J. Neurosci..

[117] Goldman S.A., Kuypers N.J. (2015). How to make an oligodendrocyte. Development (Cambridge, England).

[118] Haines J.D., Fulton D.L., Richard S., Almazan G. (2015). p38 mitogen-activated protein kinase pathway regulates genes during proliferation and differentiation in oligodendrocytes. PLoS One.

[119] Ahmed S., Gan H.T., Lam C.S., Poonepalli A., Ramasamy S., Tay Y., Tham M., Yu Y.H. (2009). Transcription factors and neural stem cell self-renewal, growth and differentiation. Cell Adh. Migr..

[120] Wang J., Pol S.U., Haberman A.K., Wang C., O’Bara M.A., Sim F.J. (2014). Transcription factor induction of human oligodendrocyte progenitor fate and differentiation. Proc. Natl. Acad. Sci. U. S. A..

[121] Wegner M. (2000). Transcriptional control in myelinating glia: the basic recipe. Glia.

[122] Menassa D.A., Gomez-Nicola D. (2018). Microglial dynamics during human brain development. Front. Immunol..

[123] Lenz K.M., Nelson L.H. (2018). Microglia and beyond: innate immune cells as regulators of brain development and behavioral function. Front. Immunol..

[124] Lima F.R.S., da Fonseca A.C., Faria G.P., Dubois L.G., Alves T.R., Faria J., Moura Neto V., Ulrich H. (2010). The origin of microglia and the development of the brain. Perspectives of Stem Cells.

[125] Arthur-Farraj P., Wanek K., Hantke J., Davis C.M., Jayakar A., Parkinson D.B., Mirsky R., Jessen K.R. (2011). Mouse schwann cells need both NRG1 and cyclic AMP to myelinate. Glia.

[126] Monk K.R., Naylor S.G., Glenn T.D., Mercurio S., Perlin J.R., Dominguez C., Moens C.B., Talbot W.S. (2009). A G protein-coupled receptor is essential for Schwann cells to initiate myelination. Science (New York, N.Y.).

[127] Doddrell R.D., Dun X.P., Moate R.M., Jessen K.R., Mirsky R., Parkinson D.B. (2012). Regulation of Schwann cell differentiation and proliferation by the Pax-3 transcription factor. Glia.

[128] Monk K.R., Feltri M.L., Taveggia C. (2015). New insights on Schwann cell development. Glia.

[129] Kiryushko D., Korshunova I., Berezin V., Bock E. (2006). Neural cell adhesion molecule induces intracellular signaling via multiple mechanisms of Ca2+ homeostasis. Mol. Biol. Cell.

[130] Patel B.N. (2002). D.L. Van Vactor, axon guidance: the cytoplasmic tail. Curr. Opin. Cell Biol..

[131] Yuan X.B., Jin M., Xu X., Song Y.Q., Wu C.P., Poo M.M., Duan S. (2003). Signalling and crosstalk of Rho GTPases in mediating axon guidance. Nat. Cell Biol..

[132] Stappert L., Roese-Koerner B., Brüstle O. (2015). The role of microRNAs in human neural stem cells, neuronal differentiation and subtype specification. Cell Tissue Res..

[133] Johnston R.J., Chang S., Etchberger J.F., Ortiz C.O., Hobert O. (2005). MicroRNAs acting in a double-negative feedback loop to control a neuronal cell fate decision. Proc. Natl. Acad. Sci. U. S. A..

[134] Johnston R.J., Hobert O. (2003). A microRNA controlling left/right neuronal asymmetry in Caenorhabditis elegans. Nature.

[135] Bonev B., Pisco A., Papalopulu N. (2011). MicroRNA-9 reveals regional diversity of neural progenitors along the anterior-posterior axis. Dev. Cell.

[136] Bonev B., Stanley P., Papalopulu N. (2012). MicroRNA-9 modulates Hes1 ultradian oscillations by forming a double-negative feedback loop. Cell Rep..

[137] Shibata M., Kurokawa D., Nakao H., Ohmura T., Aizawa S. (2008). MicroRNA-9 modulates Cajal-Retzius cell differentiation by suppressing Foxg1 expression in mouse medial pallium. J. Neurosci..

[138] Shibata M., Nakao H., Kiyonari H., Abe T., Aizawa S. (2011). MicroRNA-9 regulates neurogenesis in mouse telencephalon by targeting multiple transcription factors. J. Neurosci..

[139] de Chevigny A., Coré N., Follert P., Gaudin M., Barbry P., Béclin C., Cremer H. (2012). miR-7a regulation of Pax6 controls spatial origin of forebrain dopaminergic neurons. Nat. Neurosci..

[140] Anderegg A., Lin H.P., Chen J.A., Caronia-Brown G., Cherepanova N., Yun B., Joksimovic M., Rock J., Harfe B.D., Johnson R., Awatramani R. (2013). An Lmx1b-miR135a2 regulatory circuit modulates Wnt1/Wnt signaling and determines the size of the midbrain dopaminergic progenitor pool. PLoS Genet..

[141] Leucht C., Stigloher C., Wizenmann A., Klafke R., Folchert A., Bally-Cuif L. (2008). MicroRNA-9 directs late organizer activity of the midbrain-hindbrain boundary. Nat. Neurosci..

[142] Decembrini S., Bressan D., Vignali R., Pitto L., Mariotti S., Rainaldi G., Wang X., Evangelista M., Barsacchi G., Cremisi F. (2009). MicroRNAs couple cell fate and developmental timing in retina. Proc. Natl. Acad. Sci. U. S. A..

[143] La Torre A., Georgi S., Reh T.A. (2013). Conserved microRNA pathway regulates developmental timing of retinal neurogenesis. Proc. Natl. Acad. Sci. U. S. A..

[144] Asli N.S., Kessel M. (2010). Spatiotemporally restricted regulation of generic motor neuron programs by miR-196-mediated repression of Hoxb8. Dev. Biol..

[145] Chen J.A., Huang Y.P., Mazzoni E.O., Tan G.C., Zavadil J., Wichterle H. (2011). Mir-17-3p controls spinal neural progenitor patterning by regulating Olig2/Irx3 cross-repressive loop. Neuron.

[146] Luxenhofer G., Helmbrecht M.S., Langhoff J., Giusti S.A., Refojo D., Huber A.B. (2014). MicroRNA-9 promotes the switch from early-born to late-born motor neuron populations by regulating Onecut transcription factor expression. Dev. Biol..

[147] Otaegi G., Pollock A., Hong J., Sun T. (2011). MicroRNA miR-9 modifies motor neuron columns by a tuning regulation of FoxP1 levels in developing spinal cords. J. Neurosci..

[148] Dotti C.G., Esteban J.A., Ledesma M.D. (2014). Lipid dynamics at dendritic spines. Front. Neuroanat..

[149] McCrea H.J., De Camilli P. (2009). Mutations in phosphoinositide metabolizing enzymes and human disease. Physiology (Bethesda, Md.).

